# Supplementary figures and images for: Production of a rabbit monoclonal antibody for highly sensitive detection of citrus mosaic virus and related viruses
Source: PLoS One. 2020 Apr 15;15(4):e0229196. doi: 10.1371/journal.pone.0229196 (PMC7159214; doi:10.1371/journal.pone.0229196)

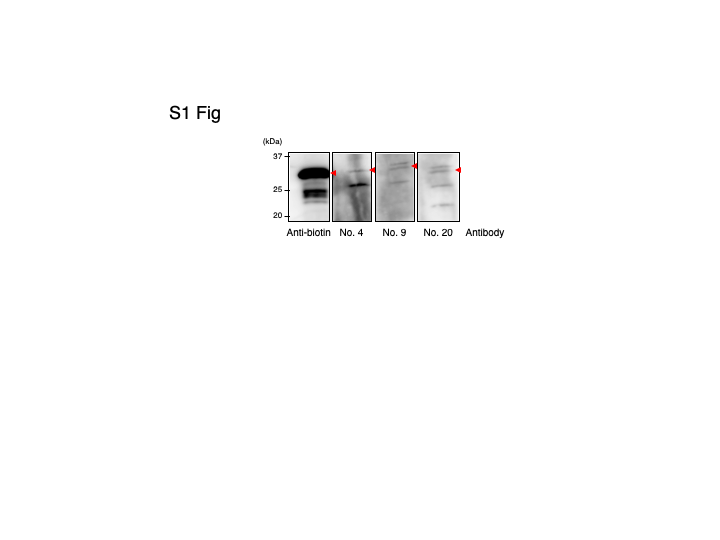

Supplement: S1 Fig — Three mAbs were used for detection of biotinylated GST-fused CiMV coat protein fragment by immunoblotting. Arrowheads indicate GST-fused CiMV coat protein fragment. (TIFF) [file pone.0229196.s001.tiff]

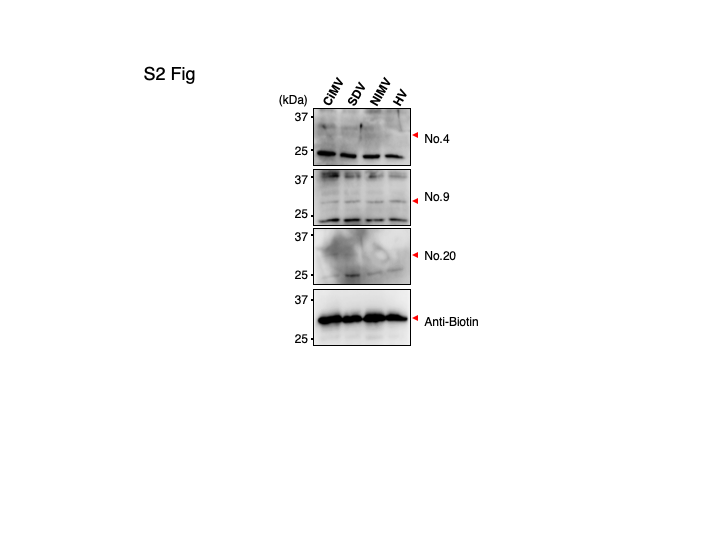

Supplement: S2 Fig — Three mAbs were used for detection of four different biotinylated SDV-like virus peptide-fused GST proteins synthesized using the cell-free system. Arrowheads indicate SDV-like virus peptide-fused GST proteins. (TIFF) [file pone.0229196.s002.tiff]

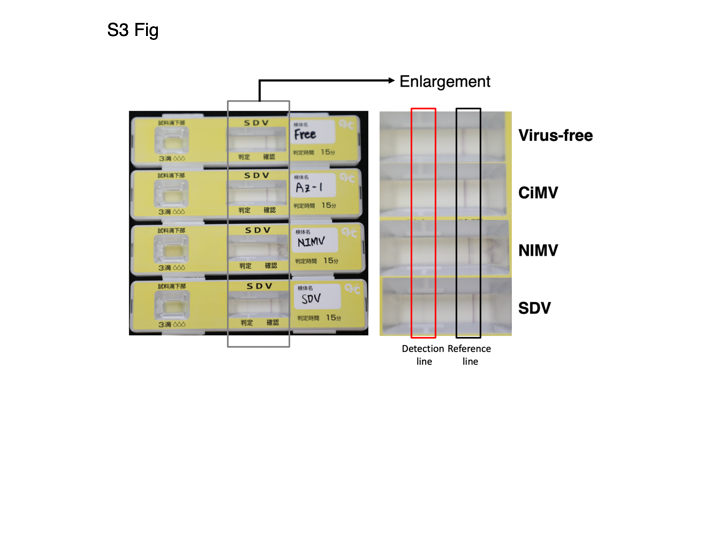

Supplement: S3 Fig — Leaves of virus-infected rough lemon were applied to detection assay. Detection of viruses using an SDV detection kit (SDV Chromato, Mizuho Medy Co., Ltd., Tosu, Japan) was performed according to the manufacturer’s instruction. (TIFF) [file pone.0229196.s003.tiff]

Fig. 4B

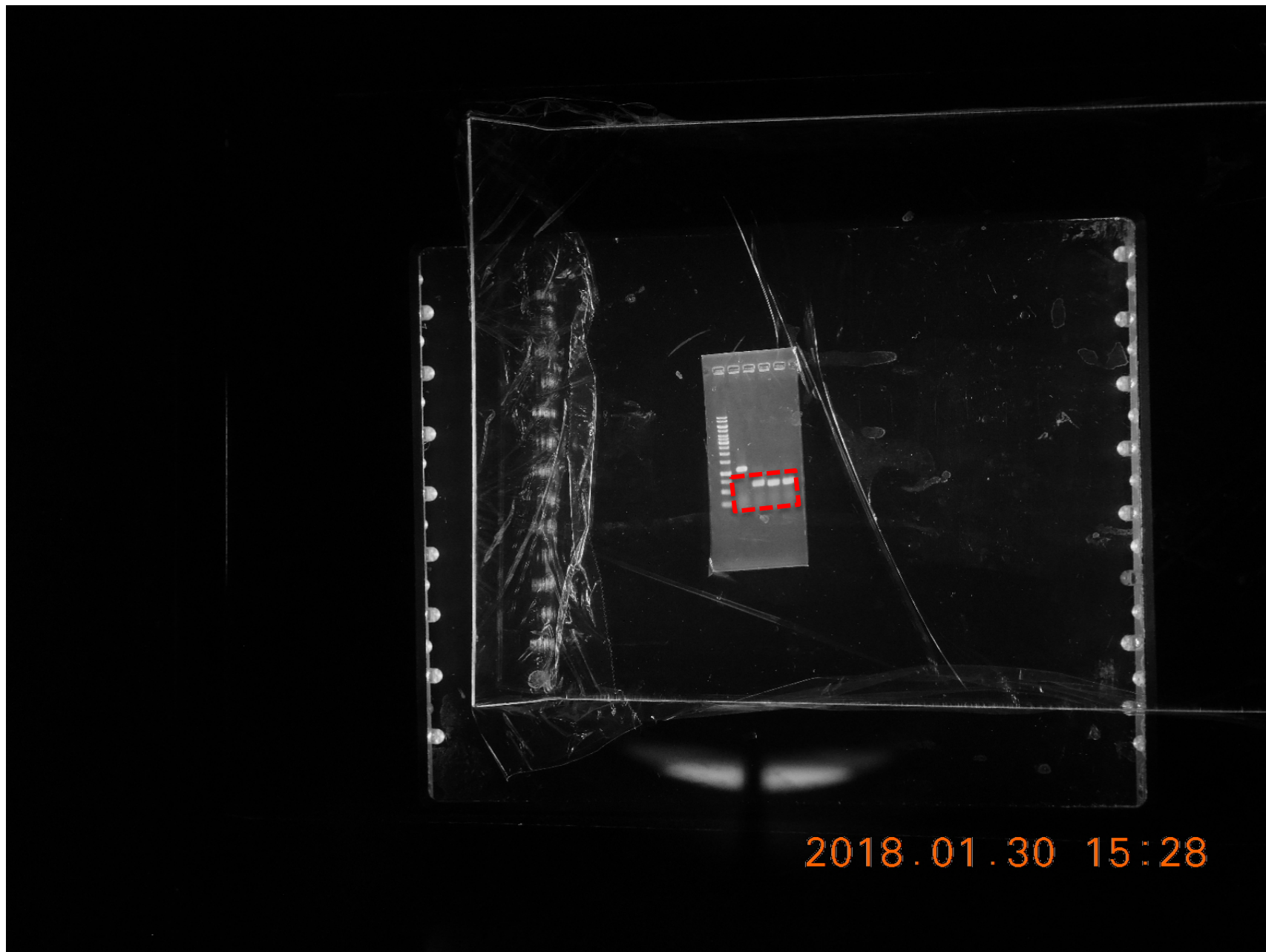

S1 Fig.

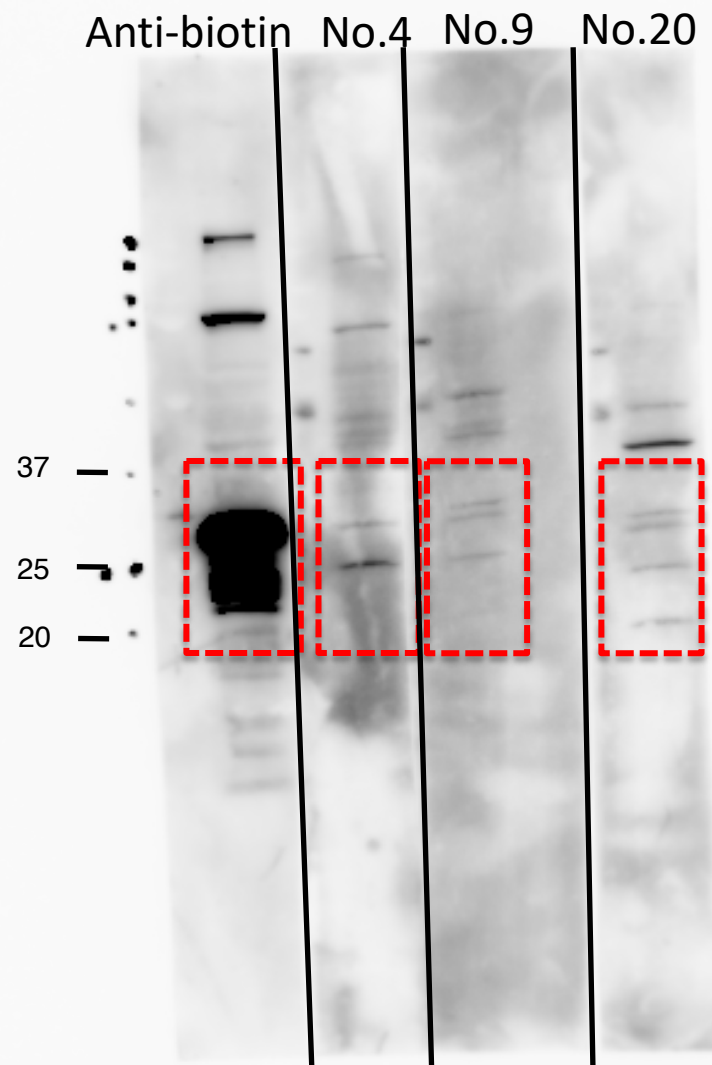

S2 Fig.

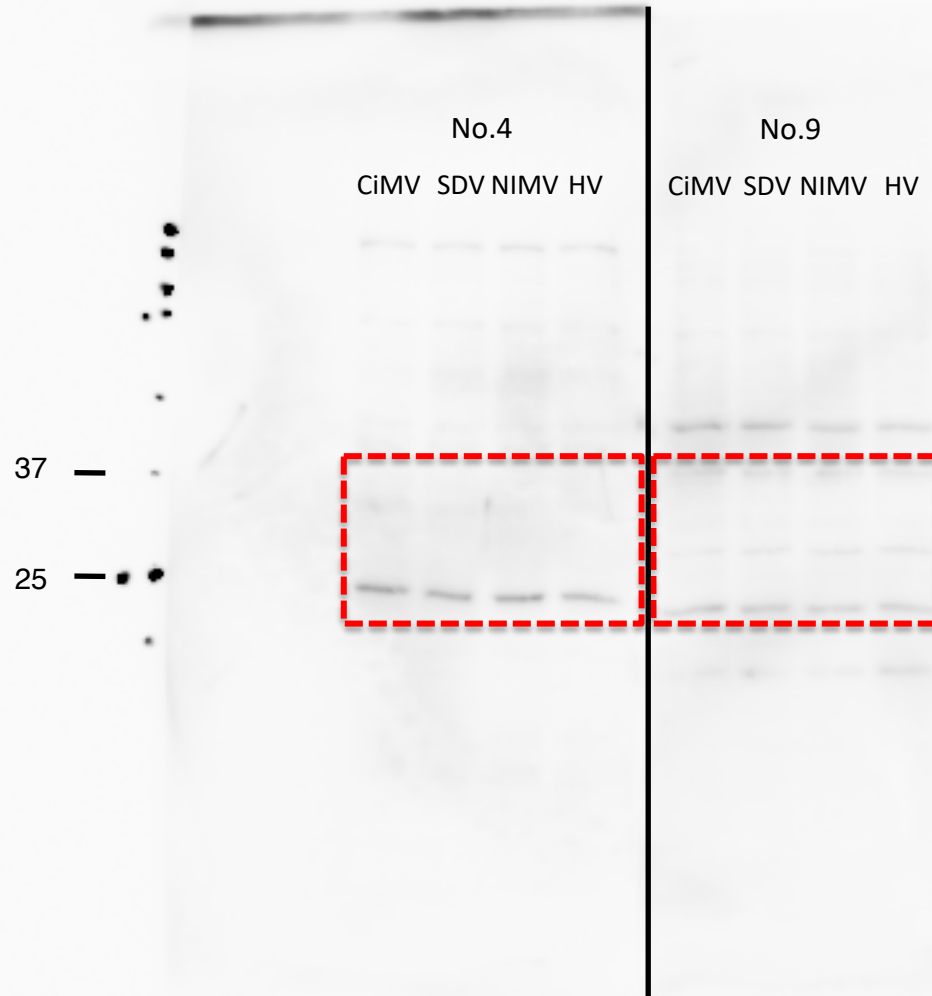

S2 Fig.

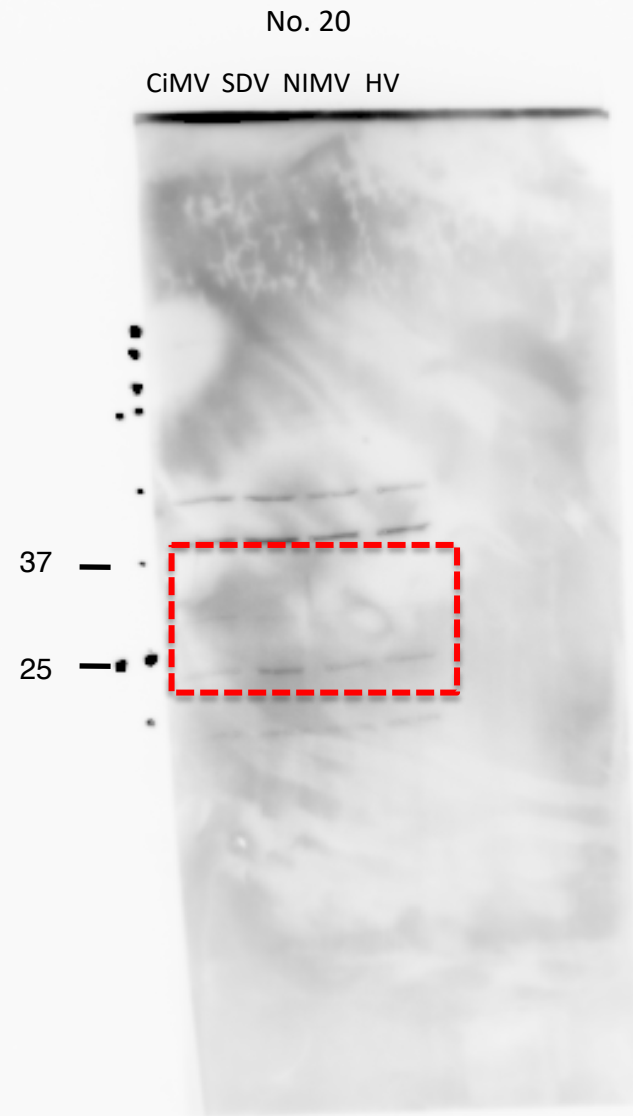

S2 Fig.

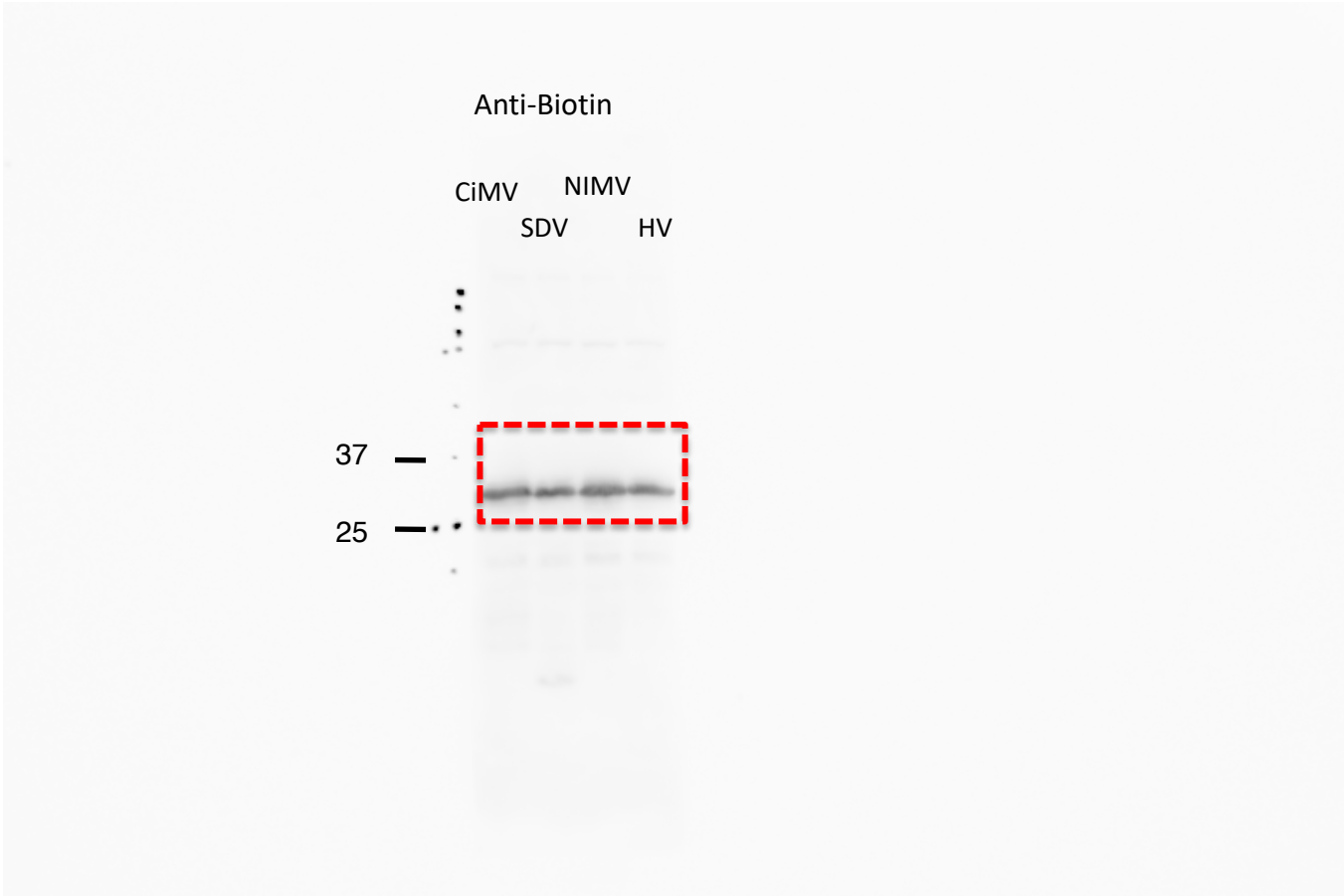

Supplement: S1 Raw image — (PDF) [file pone.0229196.s006.pdf]
