# Supplementary material for: Production of a rabbit monoclonal antibody for highly sensitive detection of citrus mosaic virus and related viruses
Source: PLoS One. 2020 Apr 15;15(4):e0229196. doi: 10.1371/journal.pone.0229196 (PMC7159214; doi:10.1371/journal.pone.0229196)
Supplement: S1 File — (DOCX) [file pone.0229196.s005.docx]

S1 File

**Method of immunoblot analysis**

After separation by SDS-PAGE using 12.5% acrylamide gel, proteins were transferred to polyvinylidene difluoride membranes (Merck Millipore, Burlington, MA). After blocking with 5% skim milk solubilized in a TBST buffer containing 20 mM Tris-HCl (pH 7.5), 150 mM NaCl, and 0.05% Tween 20 for 1 h, the membrane was treated for 1 h by one of the following primary antibodies at the appropriate concentration: anti-CiMV coat protein antibodies (No. 4, No. 9, or No. 20 (0.001 µg/ml)) or HRP-conjugated anti-biotin antibody (1:10,000 dilution; Sigma-Aldrich Japan, Tokyo, Japan). After washing thrice with TBST buffer, the membrane was treated with the secondary antibody HRP-conjugated anti-rabbit IgG antibody (1:10000 dilution; GE Healthcare Japan, Hino, Japan), except when primary antibody was already conjugated to HRP. After the membrane was washed with TBST buffer for three times, the antibodies were detected with ImmunoStar® LD (FUJIFILM Wako Pure Chemical Corporation, Osaka, Japan) using an ImageQuant LAS 4000 imager (GE Healthcare Japan, Hino, Japan).
