# Supplementary material for: Production of a rabbit monoclonal antibody for highly sensitive detection of citrus mosaic virus and related viruses
Source: PLoS One. 2020 Apr 15;15(4):e0229196. doi: 10.1371/journal.pone.0229196 (PMC7159214; doi:10.1371/journal.pone.0229196)
Supplement: Attachment — Submitted filename: PONE-D-19-23810_review.docx [file pone.0229196.s007.docx]

**Production of a rabbit monoclonal antibody for highly sensitive detection of citrus mosaic virus and related viruses by Miyoshi et al (Manuscript ID #PONE-D-19-23810; PLOS ONE).**

The authors have reported characterization of rabbit monoclonal antibody for sensitive detection of citrus mosaic virus (CiMV) and related viruses. The manuscript is well written with proper introduction and need for this study. However, there are several shortcomings as listed below.

1. Have you compared the sensitivity of commercially available kit (by Kusano et al) against novel CiMV isolates in your studies? Data has to be included to compare sensitivity of the mAbs identified in this paper.
2. How similar are these viruses with respect to viral genome (CiMV, novel CiMV isolates and SDV-related viruses), discuss in the paper.
3. What is the difference between Fig 1C and 1D? Did you include negative control (DHFR) in Fig 1C? Fig 1 legend is not clear on CiMV coat protein/ peptide, needs to be modified.
4. Fig 1E, although there is corresponding staining as indicated by arrowheads, there are additional bands – is it expected?
5. AlphaScreen data shows differential sensitivity for the various peptides from SDV-like viruses, Fig 3B. Is there a statistical difference between test groups vs. negative control (DHFR)? Also, what does the error bars means – include details in the figure legend.
6. Include corresponding amino acid residue numbers in Fig 3A.
7. It is not clear about the specificity mAbs in Fig 3C, except for mAb No. 9 (Lines 277 to 279).
8. Provide reference for statements in the Introduction section (lines 67 to 70).
9. Perform statistical analysis for the ELISA data in Fig 4C, provide details on number of experiments including replicates involved.
10. How many experiments involved in Fig 4D? Provide quantitated dot-blot analysis based on staining density – this analysis help better understand sensitivity of mAb No. 20.
